# Supplementary material for: Center-surround interactions underlie bipolar cell motion sensitivity in the mouse retina
Source: Nat Commun. 2022 Sep 26;13:5574. doi: 10.1038/s41467-022-32762-7 (PMC9513071; doi:10.1038/s41467-022-32762-7)
Supplement: Supplementary file 1 — Supplementary Information [file 41467_2022_32762_MOESM1_ESM.pdf]

Supplemental Figures

Supplementary Fig. 1: related to Figure 1
Supplementary Fig. 2: related to Figure 1
Supplementary Fig. 3: related to Figure 2
Supplementary Fig. 4: related to Figure 2
Supplementary Fig. 5: related to Figure 2
Supplementary Fig. 6: related to Figure 2
Supplementary Fig. 7: related to Figure 6 and 7
Supplementary Fig. 8: related to Figure 5
Supplementary Fig. 9: related to Figure 6
Supplementary Fig. 10: related to Figure 6
Supplementary Fig. 11: related to Figure 6 and 7

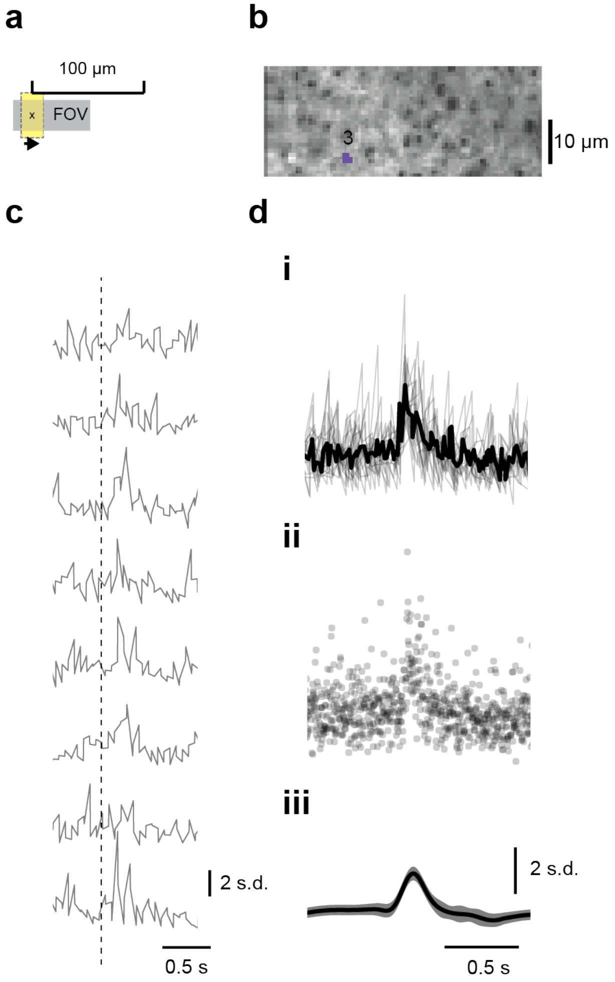

Supplementary Fig. 1. Gaussian process modeling of BC terminal responses, related to Figure 1.
(a) The moving bar stimulus as in **Fig. 1**.
(b) ROI 3 from **Fig. 1**
(c) Z-scored example single trials in response to the stimulus in (a).
(d) Comparison of averaging and Gaussian Process prediction. (i) Single trials (gray) overlaid with the binned average taken at 63 Hz. (ii) Scatter plot of all measurements from all trials. (iii) Gaussian process prediction. Grey shading is 3 s.d. around the estimated mean.

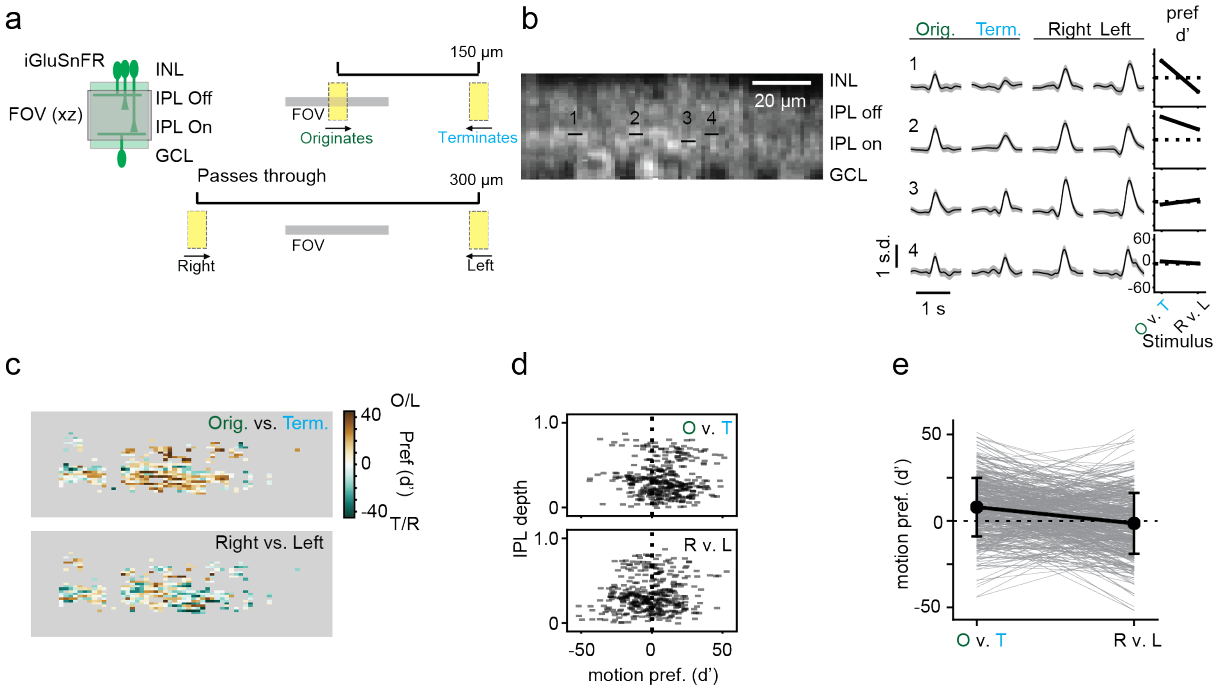

Supplementary Fig. 2. Bipolar cell responses to moving bar stimuli across the inner plexiform layer, related to Figure 1.

(a) Left: iGluSnFR is ubiquitously expressed in retinal neurons, including in the cells of the inner plexiform layer (IPL, green region). INL, inner nuclear layer; GCL, ganglion cell layer; FOV, field of view. Right: Moving bars (yellow; 20 x 40 µm), either originating from (green, O), terminating in (cyan, T) or passing through (black, R or L) the imaging FOV, presented to the retina. All objects to scale.

(b) Example ROIs (black regions, numbered) in the On layer of the IPL overlaid with s.d. of the imaged field and their responses to the stimuli in (a) as predicted using Gaussian Process modeling. Grey shading is 3 s.d. Rightmost column: motion preference (d') for each stimulus pair. Positive values represent a preference for originating or leftward motion.

(c) The motion preference (d') for all ROIs in the field.

(d) (d') as a function of IPL depth for all ROIs in the field.

(e) Motion preference (d') for each ROI in the population for originating vs. terminating motion or motion passing through. Black, mean ± s.d.; grey, individual ROIs. The two conditions are significantly different (originating vs. terminating, $d' = 8.3 \pm 16.9$; passing through, $d' = -1.5 \pm 17.5$; two-sided paired T-test, $T = 8.867$, $p = 2.95e-17$). Sample size in is 381 ROIs/ 1 field/ 1 mouse.

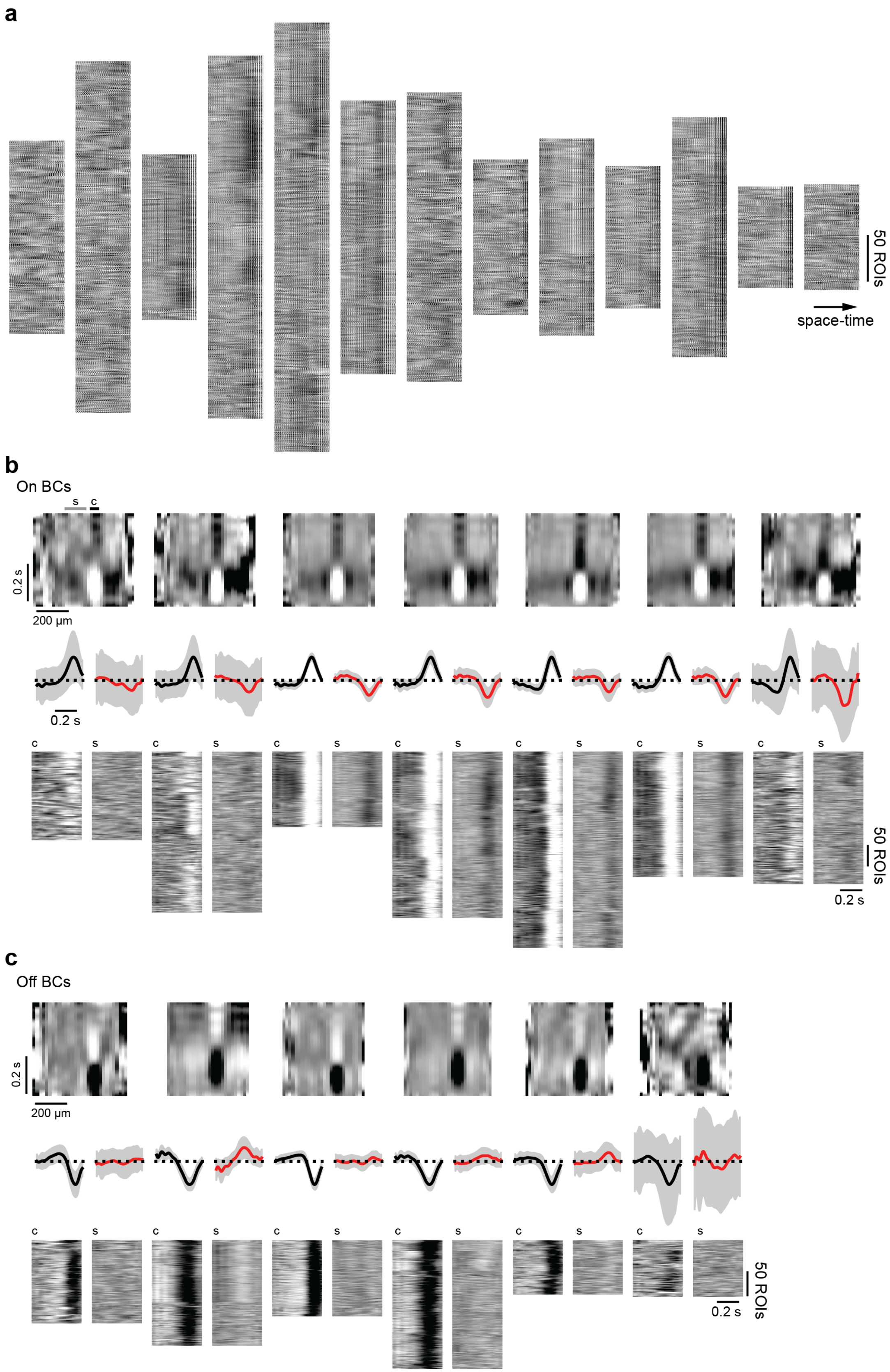

Supplementary Fig. 3. Clustered bipolar cell receptive fields and in-cluster variability, related to Figure 2

(a) Images of individual ROIs' RFs, cropped and flattened in space-time, sorted by cluster. Clusters are ordered by IPL depth.

(b) RFs of On BC clusters. First row: average receptive field of each cluster, showing additional edge regions that were not

used for modeling. Data is noisier because sample sizes are lower for more peripheral regions. “c” is center region used for extracting time kernels, “s” is surround region. Second row: center (black) and surround (red) time kernels for each cluster, normalized to the peak of the center response. Grey is s.d. Third row: center (“c”) and surround (“s”) time kernels for each individual ROI, sorted by cluster.
(c) RFs of Off BC clusters, conventions as in (b).

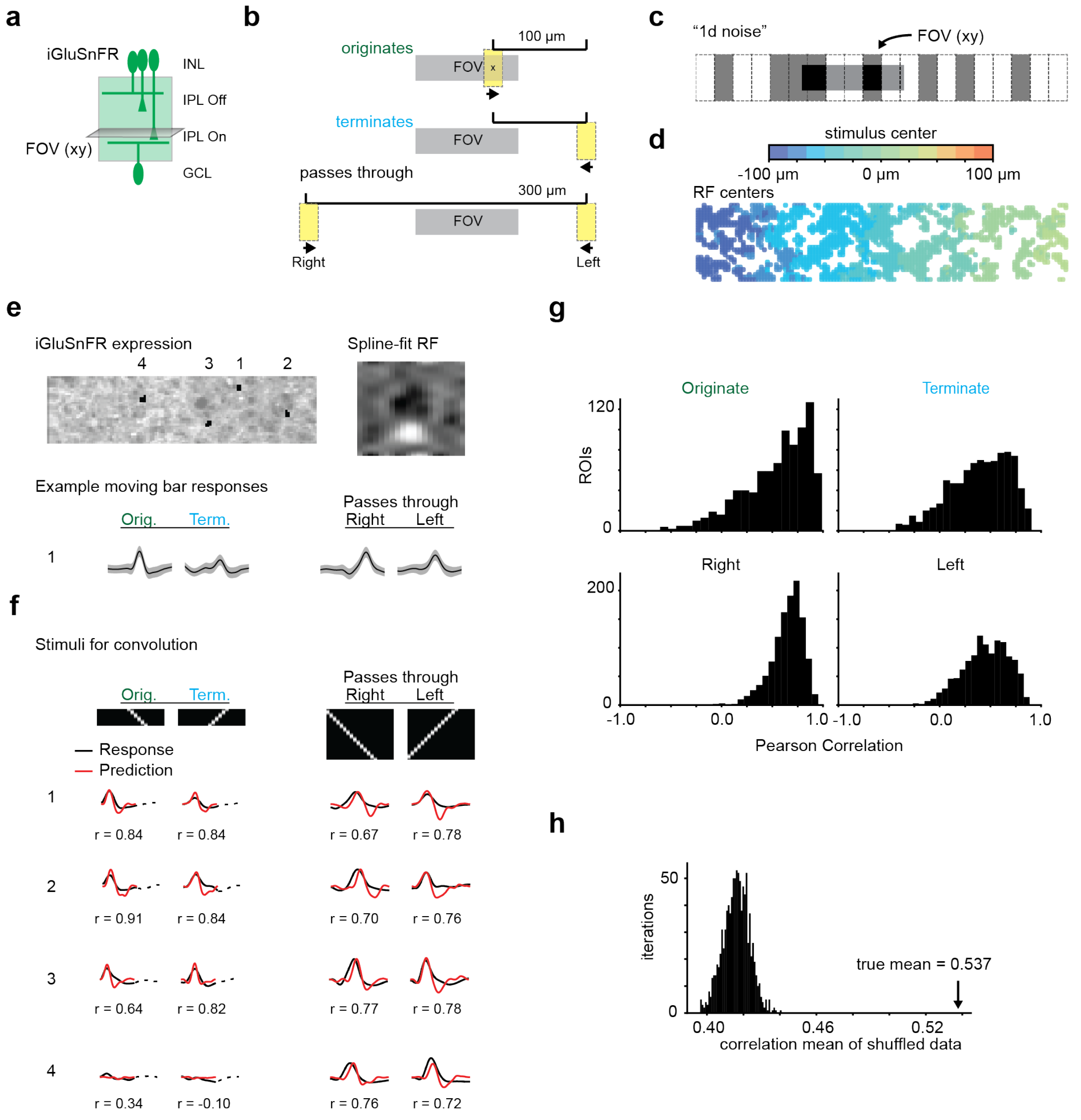

Supplementary Fig. 4. Validation of linear convolution model for predicting bipolar cell responses, related to Figure 2

(a) Diagram showing XY scan field (FOV, "field of view") location in the retina expressing iGluSNFR (green).
(b) Moving bar stimulus shown to scale with the FOV (for details, see Figure 1).
(c) Noise stimulus shown to scale with the FOV.
(d) The RF centers determined from noise for this FOV, relative to the stimulus center. Here, the stimulus center was located near the right end of the FOV.
(e) Left: standard deviation image of the FOV overlaid with example ROI locations. Right: spline receptive field for ROI 1. Bottom: example Gaussian Process predictions for the moving bar responses of ROI 1.
(f) Convolution of RFs with moving bar stimuli to predict BC responses. Four example ROIs are shown at different locations in the FOV. "Response" (black) refers to the Gaussian Process prediction of the moving bar response (determined as in **Fig. 1**). "Prediction" (red) is the result of the convolution of moving bar stimuli with that ROI's RF (determined as in **Fig. 2**). Pearson correlation is shown below each trace, $p < 0.01$ for all traces. Example ROI 4 shows low correlations because the ROI was outside of the stimulated region for the originating/terminating bar. ROIs in these locations were excluded from further analysis.
(g) Histograms of the Pearson correlations between moving bar responses to each stimulus condition and model predictions for ROIs from 3 fields/2 mice.
(h) For each distribution in (g), we performed a permutation test. We shuffled the moving bar responses between ROIs and recalculated the mean of the resulting correlation distribution. The distribution of means for 1,000 iterations is shown in here. The arrow shows the true distribution mean for this stimulus condition (originating motion). For each of the distributions in (g), the distribution mean was significantly different by permutation test with $p<0.01$.

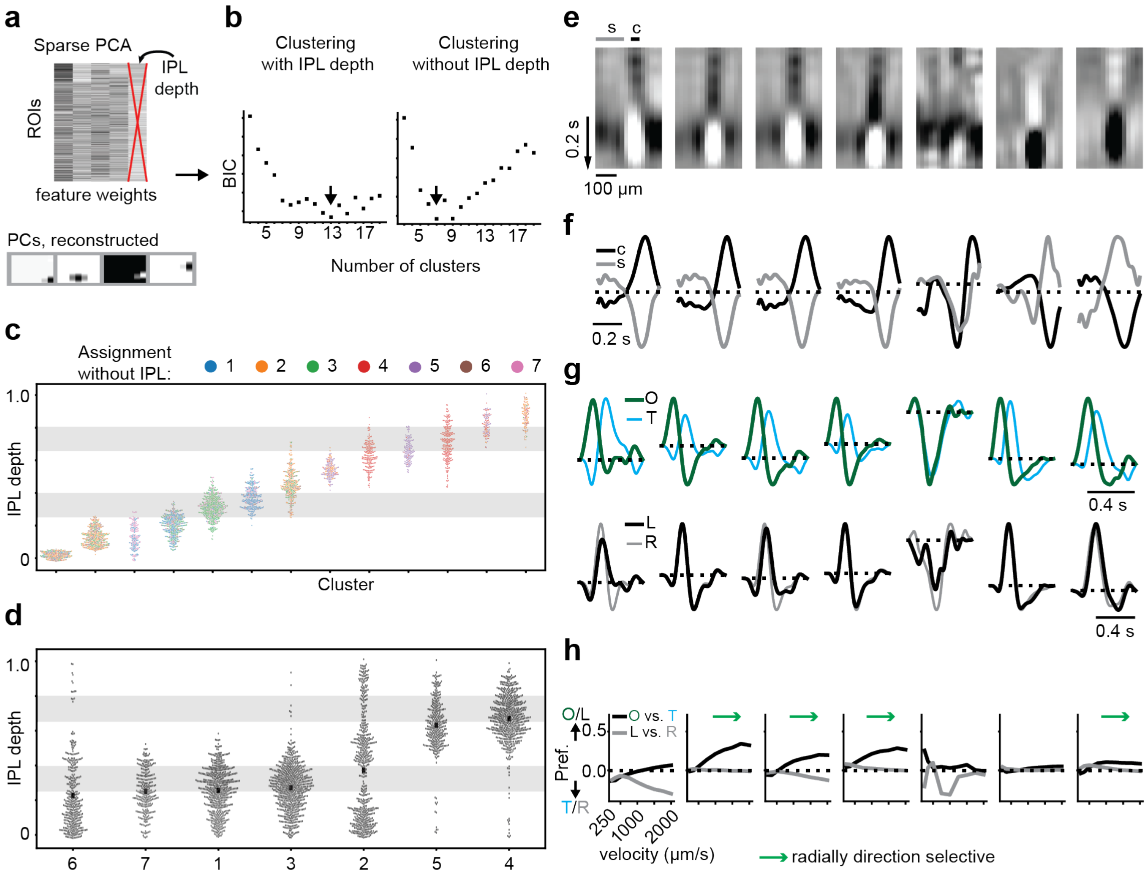

Supplementary Fig. 5. Alternative bipolar cell clustering without using the IPL as a feature, related to Figure 2

(a) Top: feature weights for the 4 components from sparse PCA. The fifth feature in **Fig. 2** (red cross) was the IPL depth of the ROI and was excluded as a feature to test for its influence on the clustering results.

(b) Mixture of Gaussian clustering was performed and the Bayesian information criterion (BIC) was used to select the number of clusters. The resulting minimum when the clustering used the IPL as a feature was 13 clusters; when the IPL depth was excluded, the minimum was 7 clusters.

(c) Cluster assignment with the IPL depth as a feature of each ROI plotted against IPL depth. Colors were assigned according to the cluster assignment from clustering without IPL depth as a feature. Clusters were reordered by average IPL depth. Grey regions: approximate ChAT bands (the dendritic plexuses of the SACs as an IPL landmark). In some cases, including IPL depth as a feature resulted in a cluster being split into multiple clusters (i.e. cluster 4), in other cases including IPL depth re-mixed the ROI assignments (i.e. cluster 1).

(d) Cluster assignment without the IPL depth as a feature of each ROI plotted against IPL depth. Cluster numbers are arbitrary, but consistent between (c) and (d).

(e) Average RF of each cluster. “c” and “s” show the regions used to calculate the spatial average of the center and surround in (f).

(f) Average temporal RFs taken from the center (“c”) and surround (“s”) regions indicated in (e), normalized to their respective peaks.

(g) Modeled responses to motion (velocity 1,000 µm/s) for each cluster for stimuli originating (green, “O”) or terminating (cyan, “T”) in the RF center or passing over the full RF (black, “L”, grey “R”).

h) Radial direction preference as a function of velocity for each cluster for the originating vs. terminating or left vs. right stimuli. Even with fewer clusters, we observed diversity in the predicted radial direction-selectivity (rDS) between clusters.

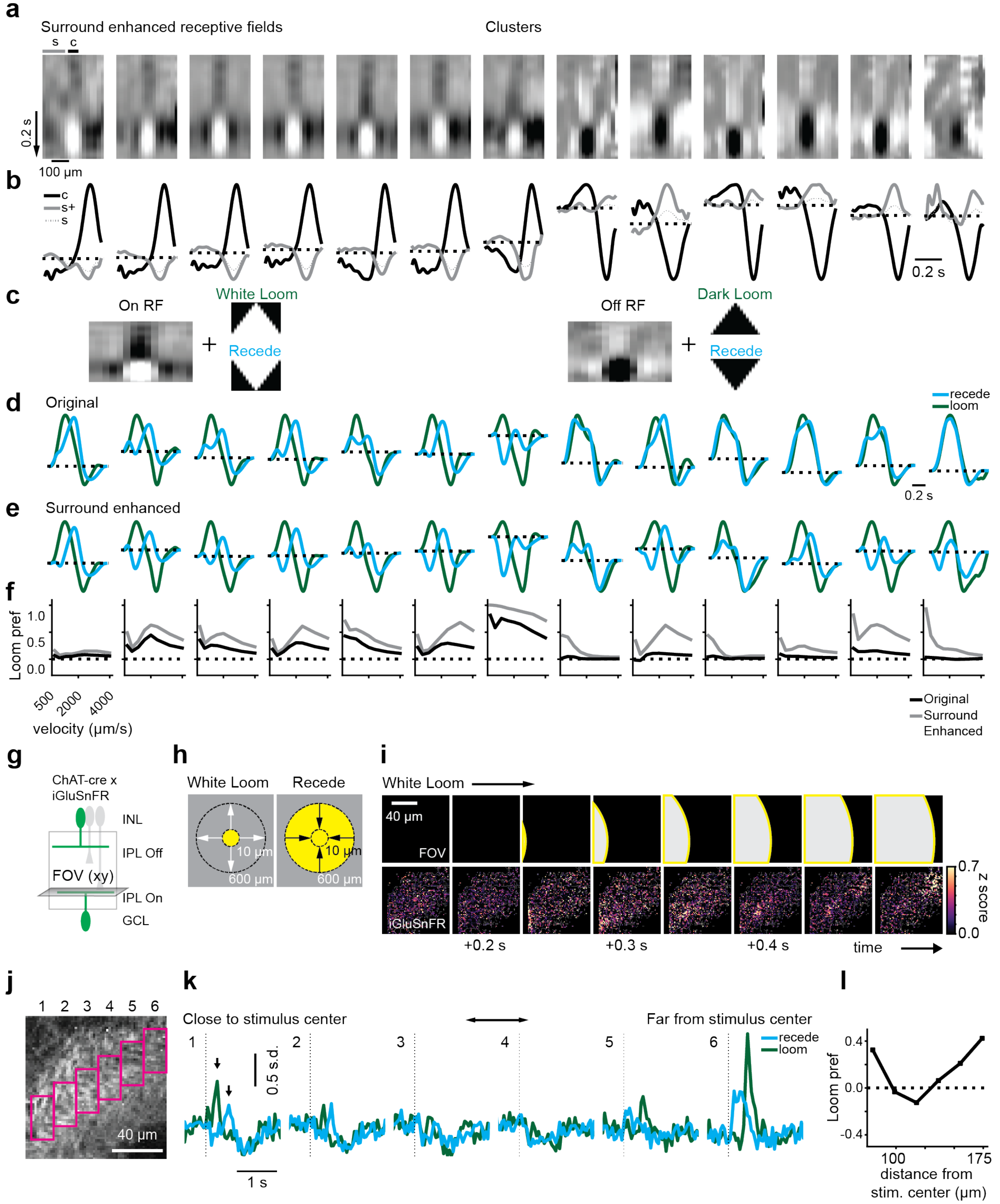

Supplementary Fig. 6. Modeling reveals differing looming preference of bipolar cell clusters, related to Figure 2.

(a) Cluster average RFs with the surround strength enhanced. On BCs were enhanced 150%, while Off BCs were enhanced 300%. “c” and “s” show the regions used to calculate the spatial average of the center and surround in (b).

(b) Average temporal RFs taken from the center (“c”) and surround (“s”) regions indicated in (a), showing the relative amplitude of center and surround. For surround, dotted (“s”) is the original surround, solid (“s+”) is the enhanced surround.

(c) Example RF convolved with looming (green) and receding (cyan) stimuli to model responses of BC clusters.

(d) Example modeled responses of each BC cluster to looming and receding stimuli at a stimulus velocity of 1,000 µm/s using the cluster average RFs from **Fig.2**.

(e) Example modeled responses of each BC cluster to looming and receding stimuli at a stimulus velocity of 1,000 µm/s using

the surround enhanced RFs in (a).
(f) Looming preference of each BC cluster's RF across stimulus velocities. Looming-sensitive BC clusters exhibit even stronger looming preference when the surround is strengthened (black vs. grey).
(g) Experiment to validate looming response predictions. flex-iGluSnFR was injected into ChAT-cre mice to achieve SAC-specific labeling (green).
(h) White looming and receding stimuli. For looming, a white spot (indicated in yellow) appears on a dark background with a diameter of 10 µm and expands to 600 µm at a rate of expansion of 800 µm/s, then disappears. Receding stimulus is the reverse in time. Diagram not to scale.
(i) Response of one FOV to looming stimulus. Top row: position of the stimulus in the FOV. The center of the stimulus is outside the FOV. Bottom row: montage of the average z-scored fluorescence response of glutamate sensor iGluSnFR during stimulation.
(j) Average iGluSnFR fluorescence during stimulation, showing six ROIs (pink) used to measure fluorescence responses.
(k) Mean binned fluorescence in response to each stimulus condition for the pixels in each ROI. Arrows show peak responses used to calculate the looming sensitivity index (LSI).
(l) LSI for the responses in (k) vs. the ROI distance from the stimulus center. The position closest to the center exhibits looming sensitivity predicted by our modeling in (d)-(e).

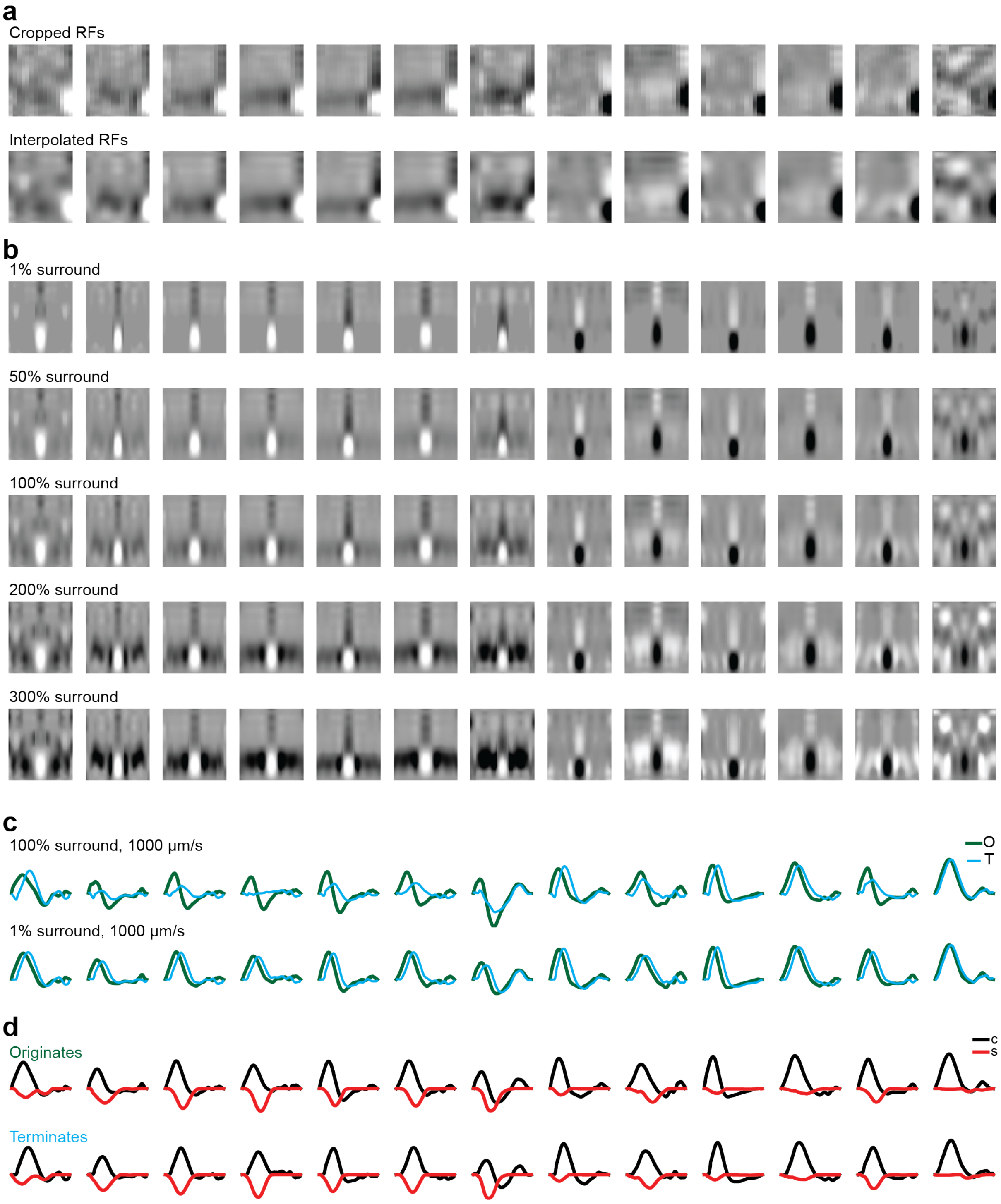

Supplementary Fig. 7. Receptive field modification for biophysical modeling and center vs. surround timing across clusters, related to Figure 6 and 7.

(a) Top: Original average RFs from clusters in **Fig. 2** were cropped to include the more-complete half of the RF. Order of clusters is the same as in **Fig. 2** (ordered based on average IPL depth from GCL to INL). Bottom: For each RF, we performed singular value decomposition (SVD). Then, we performed linear interpolation on spatial and temporal components of the SVD. Finally, the RFs were reconstructed from the first 3 spatial and temporal components to create the RFs shown.

(b) RFs prepared for biophysical SAC modeling are shown with different levels of surround strength. The RFs in (a) were reflected to create a full RF for each cluster. To change the strength of the surround, we multiplied all values that were opposite polarity to the center (i.e. negative values for On clusters, positive values for Off clusters) in the surround spatial region by a

scalar multiplier. This changed the strength of the surround while maintaining the same strength of the center region.
(c) Convolution of 1,000 μm/s moving bar stimulus for originating and terminating motion (as in **Fig. 2i**) for the 100% and 1% surround cases from (b) to model responses to moving stimuli. rDS is largely lost when the surround is minimized.
(d) Decomposition of modeled responses to originating and terminating motion. Center ("c", black) response was estimated as the response to 1% surround shown in (c). The surround ("s", red) contribution was estimated by subtracting the response modeled from RFs with 1% surround from the response predicted from 100% surround RFs. In motion-sensitive clusters, the timing of excitatory center and inhibitory surround are more offset for outward motion than for inward motion due to the center and surrounds' different temporal properties (see also **Fig. 3**).

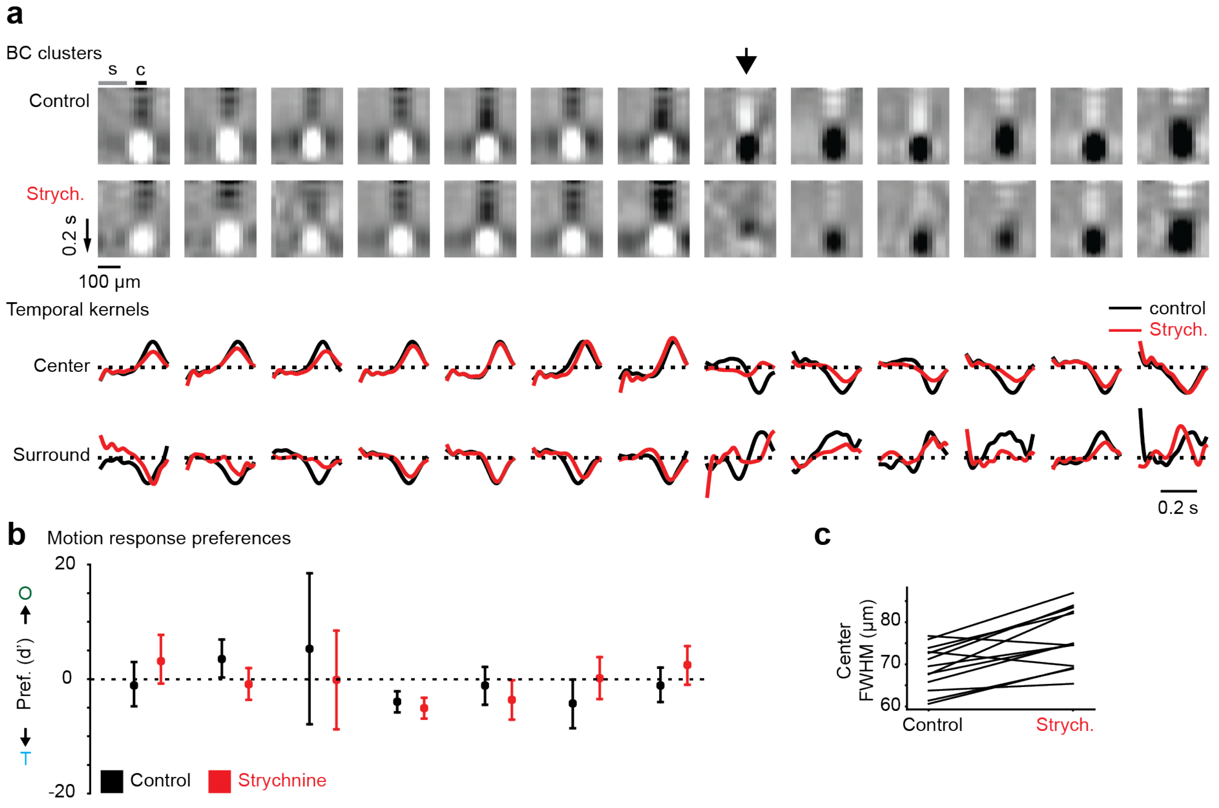

Supplementary Fig. 8. Radial direction selectivity is unchanged in the presence of glycinergic receptor blockade, related to Figure 5.

(a) Receptive fields and temporal kernels as in Fig. 5, but here in the presence of 0.5 µM strychnine. The arrow marks a cluster in which the polarity of the BC center changed from Off to On, an RF change observed in strychnine previously (1). Data from 5181 ROIs/ 5 fields/ 4 eyes/ 3 mice.

(b) rDS preference (d') for the ROIs in each cluster in control (grey) and strychnine (red). Points, the mean; error bars, the 95% confidence interval. Two-way ANOVA with repeated measures, not significant, $p = 1.0$ between clusters, $p = 0.80$ between control/drug, $p = 1.0$ for the interaction of cluster and treatment. Data from 458 ROIs/ 2 fields/ 2 eyes/ 2 mice.

(c) FWHM of the spatial RF for each cluster. Changes in the spatial extent of the center were more subtle in strychnine than in TPMPA.

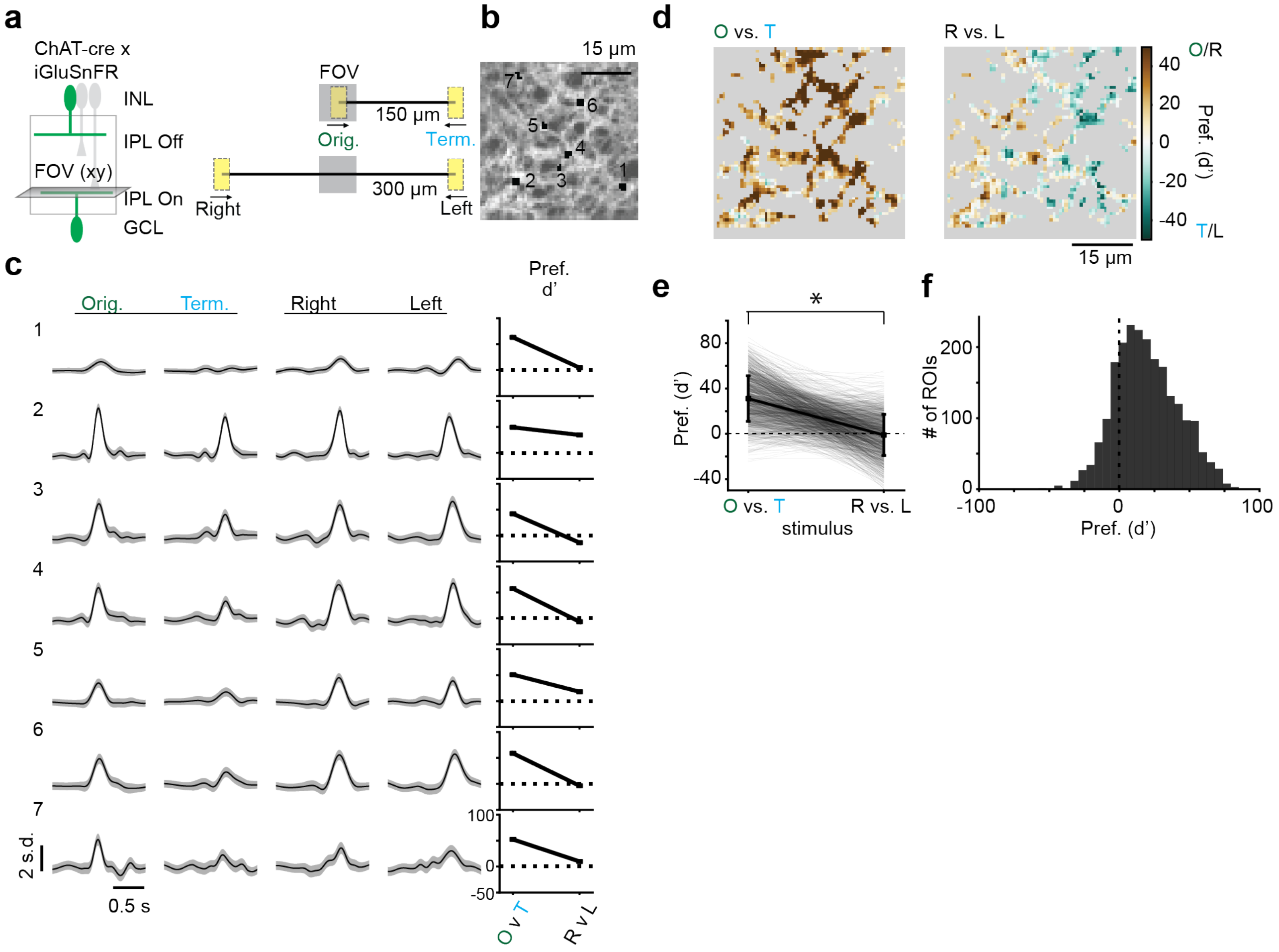

Supplementary Fig. 9. Bipolar cell inputs onto starburst amacrine cells are motion-sensitive, related to Figure 6.
(a) Left: flex-iGluSnFR injected into ChAT-cre mice to achieve SAC-specific labeling. Right: Moving bar stimulus (20 x 40 µm rectangle) moving at 500 µm/s over a distance of 150 or 300 µm to evoke responses to the origin of motion, termination of motion, or motion passing through. Diagram to scale.
(b) S.d. of the scan field showing iGluSnFR expression in SACs. Black regions/numbers: ROIs in (c).
(c) Responses predicted with Gaussian Process for each stimulus condition from (a). Numbers correspond to ROIs in (b). Grey shading is 3 s.d. Rightmost column: rDS preference (d') for originating vs. terminating motion or motion passing through in two directions.
(d) The preference (d') for all ROIs in example field for originating vs. terminating motion or left vs. rightward motion.
(e) Comparison of motion preference between originating/terminating motion and left/right motion for all ROIs in the example field (n = 1,134 ROIs). Black, mean values $\pm$ s.d.; grey, individual ROIs. Originating vs. terminating, $d' = 31.1 \pm 20.1$; right vs. left, $d' = -1.1 \pm 18.2$). Significant with $p = 6.46e-152$, Wilcoxon test, two-sided.
(f) Distribution of preference for origin vs. termination of motion for 2,225 ROIs from 2 mice. The mean of this distribution is $d' = 19.4 \pm 22.5$. Significantly different from 0, with $p = 1.91e-302$, one sample t-test.

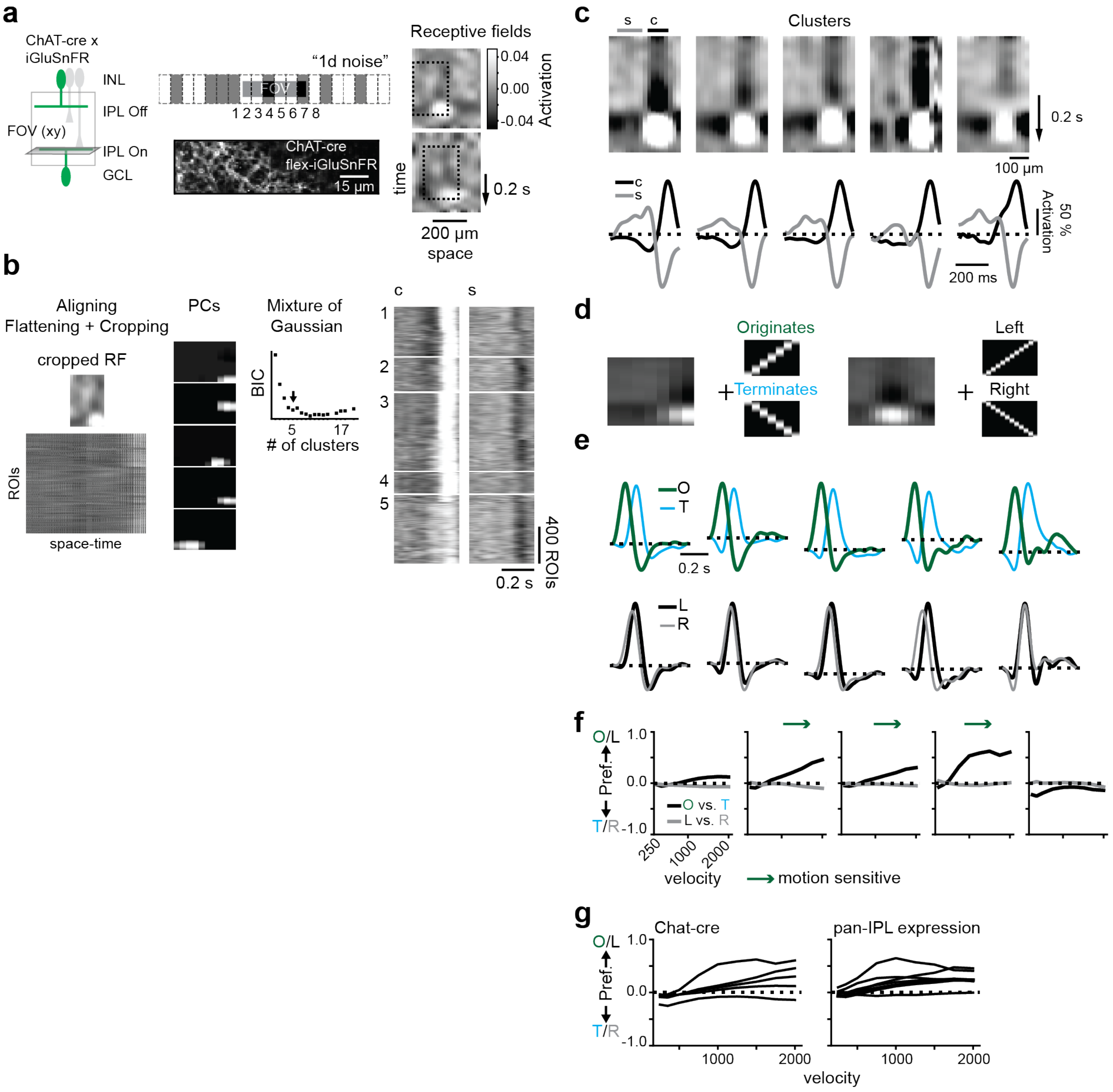

Supplementary Fig. 10. Receptive fields of bipolar inputs onto starburst amacrine cells have diverse radial direction selectivity, related to Figure 6.

(a) Left: flex-iGluSnFR injected into ChAT-cre mice to achieve SAC specific labeling. Middle: "1D noise stimulus" (top) and s.d. image of FOV (bottom) used to measure RFs from On layer SACs. Right: RFs for two example ROIs.

(b) Procedure for performing clustering of RFs similar to Fig. 2. Here, the IPL depth is not included as a feature and the optimal number of clusters was 5. Right: center and surround responses of individual ROIs in each cluster. This data set includes 2,725 ROIs from 2 mice.

(c) Top: Average RFs for each of 5 clusters. Bottom: Average temporal RFs taken from the center ("c", black) and surround ("s", gray) regions, normalized to their peaks.

(d) Convolution with half of the RF or the full RF to measure rDS or motion passing through, respectively.

(e) Modeled responses to motion (velocity 1,000 µm/s) in two directions for each cluster for originating, terminating and left vs. right motion.

(f) Preference as a function of velocity for each cluster for originating vs. terminating and left vs. right conditions.

(g) Comparison of velocity tuning curves for On BC clusters identified from mice expressing iGluSnFR only in the SACs (left, ChAT-cre) vs. ubiquitously (right, from Fig. 2).

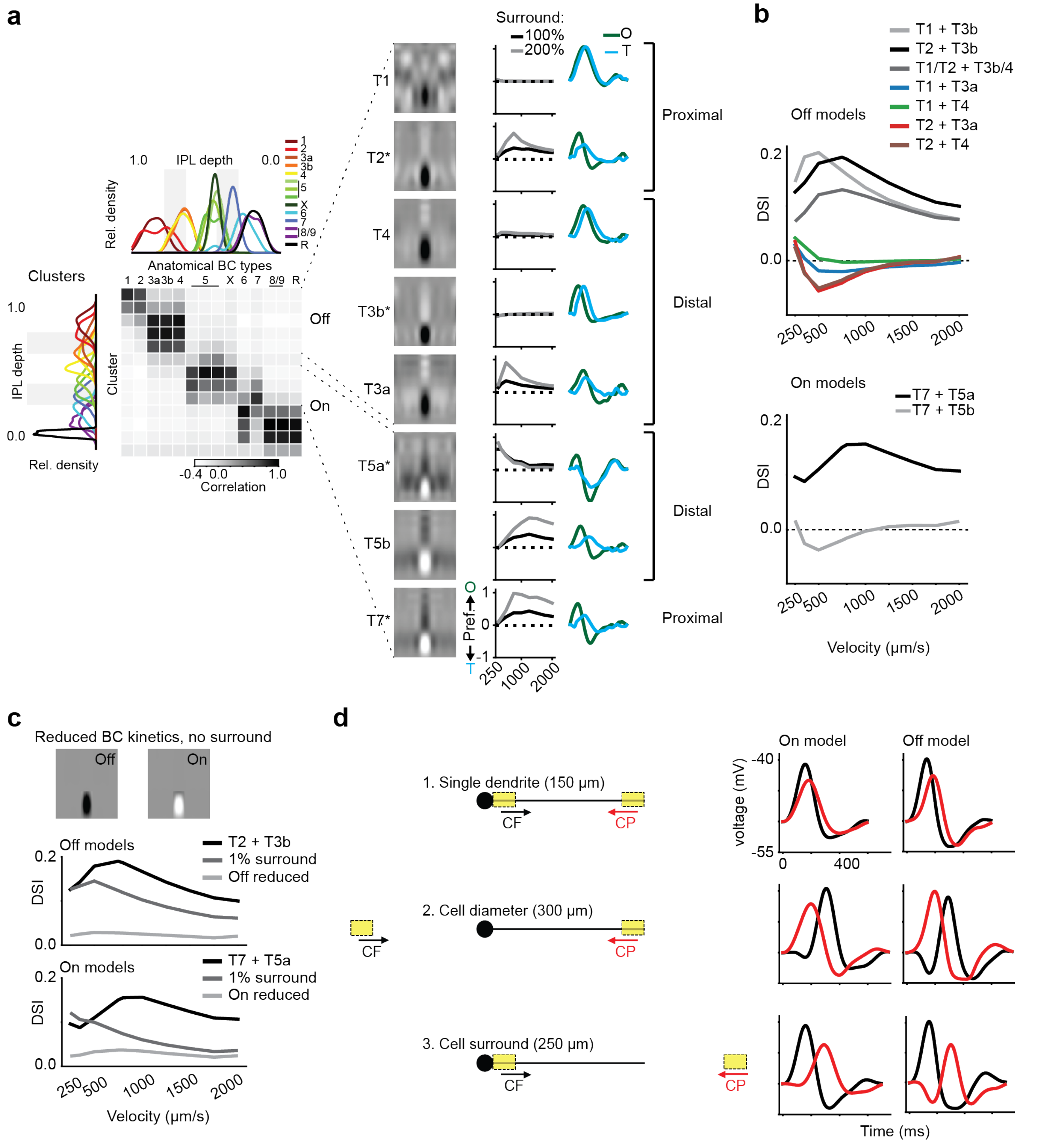

Supplementary Fig. 11. SAC models with different functional BC clusters and motion paths, related to Figure 6 and 7.

(a) Left: Pairwise correlation between IPL stratification of BC clusters identified in the current study vs. anatomically (2–4) (see also **Fig. 3**). Right: RFs of BC clusters that co-stratify with SACs (8 out of 13 identified BC clusters), their rDS across velocities, and their responses to moving bar stimuli at 1,000 µm/s with either the measured surround strength or 200% surround. Types were assigned according to IPL stratification correlation. Location-specific wiring (proximal vs. distal) of BC types along SAC dendrites (3–5) is indicated on the right. Asterisk indicates the types chosen for further modeling in **Fig. 6**.

(b) Directional tuning of SAC models using different combinations of functional BC types. Due to co-stratification, some type labels of BC clusters might need to be permuted (e.g., BC type 1 and 2, see discussion in (1)). We explored all possible combinations of one distal and one proximal type during modeling. In addition, we constructed an Off model containing four

BC types, two proximal and two distal BC types (labeled “T1/T2 + T3b/4”). Our results suggest that placing BC type 3b and 5a in the Off and On models' distal positions, respectively, is crucial for establishing a preference for CF motion. In the Off model, the inclusion of BC type 2 compared to type 1 results in a slight shift of the tuning curve towards higher velocities in the two-type model. The inclusion of further types results in a slight reduction of the DSI at low stimulus velocities. We found that BC type 2, but not type 1, conferred surround dependence of tuning, suggesting that the tuning in the type 1 model relies on a different mechanism (details not shown). Perhaps for type 1 BCs, the interaction of BC types with different temporal receptive field center properties could play a role in the preference for CF motion (3–5). Taken together, these results indicate that the properties of BC RF types determine the directional tuning of postsynaptic model SAC dendrites.

(c) Comparison of models with and without BC RF components. Top: “Reduced” BC type 2 and 7 RFs. The biphasic center response was manipulated to only include the initial response and all surround activity was removed. Middle and bottom: Comparison of the original Off and On models from **Fig. 7C** (black) with the “1% surround” models from **Fig. 7C** (dark gray) and the models using “reduced” RFs (light gray). Removing BC kinetics resulted in a reduction of the DSI at all stimulus velocities. In line with previous work (6), the SAC models with limited BC kinetics still exhibit a slight preference for CF motion. This behavior likely results from the skewed BC input distribution on the SAC dendrites, which favors sequential activation of inputs in the CF direction (6).

(d) Example depolarizations of the On and Off SAC models in response to the different spatial stimuli described in **Fig. 7** (Stimulus velocity: 1,000 µm/s). In comparison to the motion stimulus across a single dendrite, the extension of motion to the cell diameter results in a reduction of the preference for CF motion in the On model and even a switch towards a preference for CP motion in the Off model. This is due to the more symmetric activation of the BC RFs. On the other hand, the extension of the motion towards the cell surround resulted in an increase in the preference for CF motion because the surrounds of more BCs are activated in the CP direction. These findings are most prominent at high stimulus velocities (see **Fig. 7**). These results suggest that the BC input leads to a SAC RF that favors local motion stimuli originating near the SAC's soma.

References

1. Franke, K. *et al.* Inhibition decorrelates visual feature representations in the inner retina. *Nature* **542**, 439–444 (2017). URL `http://dx.doi.org/10.1038/nature21394`. `15334406`.
2. Helmstaedter, M. *et al.* Connectomic reconstruction of the inner plexiform layer in the mouse retina. *Nature* **500**, 168–174 (2013). URL `http://dx.doi.org/10.1038/nature12346`. `NIHMS150003`.
3. Kim, J. S. *et al.* Space-time wiring specificity supports direction selectivity in the retina. *Nature* **509**, 331–336 (2014). URL `http://dx.doi.org/10.1038/nature13240`.
4. Greene, M. J., Kim, J. S. & Seung, H. S. Analogous Convergence of Sustained and Transient Inputs in Parallel On and Off Pathways for Retinal Motion Computation. *Cell Reports* **14**, 1892–1900 (2016).
5. Ding, H., Smith, R. G., Poleg-Polsky, A., Diamond, J. S. & Briggman, K. L. Species-specific wiring for direction selectivity in the mammalian retina. *Nature* **535**, 105–110 (2016). URL `http://dx.doi.org/10.1038/nature18609`.
6. Vlasits, A. L. *et al.* A Role for Synaptic Input Distribution in a Dendritic Computation of Motion Direction in the Retina. *Neuron* **89**, 1317–1330 (2016). URL `http://dx.doi.org/10.1016/j.neuron.2016.02.020`.
