# Supplementary material for: Center-surround interactions underlie bipolar cell motion sensitivity in the mouse retina
Source: Nat Commun. 2022 Sep 26;13:5574. doi: 10.1038/s41467-022-32762-7 (PMC9513071; doi:10.1038/s41467-022-32762-7)
Supplement: Supplementary file 3 — Reporting Summary [file 41467_2022_32762_MOESM3_ESM.pdf]

nature portfolio

Corresponding author(s): Anna Vlasits
Thomas Euler

Last updated by author(s): Aug 06, 2022

Reporting Summary

Nature Portfolio wishes to improve the reproducibility of the work that we publish. This form provides structure for consistency and transparency in reporting. For further information on Nature Portfolio policies, see our Editorial Policies and the Editorial Policy Checklist.

Statistics

For all statistical analyses, confirm that the following items are present in the figure legend, table legend, main text, or Methods section.

n/a	Confirmed	
☐	☒	The exact sample size (*n*) for each experimental group/condition, given as a discrete number and unit of measurement
☐	☒	A statement on whether measurements were taken from distinct samples or whether the same sample was measured repeatedly
☐	☒	The statistical test(s) used AND whether they are one- or two-sided<br>*Only common tests should be described solely by name; describe more complex techniques in the Methods section.*
☒	☐	A description of all covariates tested
☐	☒	A description of any assumptions or corrections, such as tests of normality and adjustment for multiple comparisons
☐	☒	A full description of the statistical parameters including central tendency (e.g. means) or other basic estimates (e.g. regression coefficient) AND variation (e.g. standard deviation) or associated estimates of uncertainty (e.g. confidence intervals)
☐	☒	For null hypothesis testing, the test statistic (e.g. *F*, *t*, *r*) with confidence intervals, effect sizes, degrees of freedom and *P* value noted<br>*Give P values as exact values whenever suitable.*
☒	☐	For Bayesian analysis, information on the choice of priors and Markov chain Monte Carlo settings
☐	☒	For hierarchical and complex designs, identification of the appropriate level for tests and full reporting of outcomes
☐	☒	Estimates of effect sizes (e.g. Cohen's *d*, Pearson's *r*), indicating how they were calculated

Our web collection on statistics for biologists contains articles on many of the points above.

Software and code

Policy information about availability of computer code

Data collection	For image acquisition, we used custom-made software (ScanM by M. Müller and T.E.) running under IGOR Pro 6.37 for Windows (Wavemetrics). Stimuli were presented using custom Python software (QDSpy: https://github.com/eulerlab/QDSpy).
Data analysis	Data analysis was performed using Python 3 and IGOR Pro 8.0.4.2. Data were organized in a custom-written database schema using DataJoint v.0.12.8 for Python framework. Python code will be available upon publication from our GitHub repository https://github.com/eulerlab/bc-motion. Data is available from https://retinal-functomics.net/data. Additional packages: SciPy 1.5.4 scipy.org; GPy 1.9.9 https://sheffieldml.github.io/GPy; RFest (first release) https://github.com/ berenslab/RFEst; scikit-learn 0.21.3 https://scikit-learn.org; pingouin 0.3.8 https://pingouin-stats.org/; Brian2 v2.4.2 https://brian2.readthedocs.io

For manuscripts utilizing custom algorithms or software that are central to the research but not yet described in published literature, software must be made available to editors and reviewers. We strongly encourage code deposition in a community repository (e.g. GitHub). See the Nature Portfolio guidelines for submitting code & software for further information.

Data

Policy information about availability of data

All manuscripts must include a data availability statement. This statement should provide the following information, where applicable:

- Accession codes, unique identifiers, or web links for publicly available datasets
- A description of any restrictions on data availability
- For clinical datasets or third party data, please ensure that the statement adheres to our policy

Data will be available upon publication at http://www.retinal-functomics.org and on zenodo [URL to come]

Human research participants

Policy information about studies involving human research participants and Sex and Gender in Research.

Reporting on sex and gender	N/A
Population characteristics	N/A
Recruitment	N/A
Ethics oversight	N/A

Note that full information on the approval of the study protocol must also be provided in the manuscript.

Field-specific reporting

Please select the one below that is the best fit for your research. If you are not sure, read the appropriate sections before making your selection.

[x] Life sciences [ ] Behavioural & social sciences [ ] Ecological, evolutionary & environmental sciences

For a reference copy of the document with all sections, see nature.com/documents/nr-reporting-summary-flat.pdf

Life sciences study design

All studies must disclose on these points even when the disclosure is negative.

Sample size	Due to the exploratory nature of this study, no statistical methods were used to predetermine sample size. All high-quality imaging fields collected from 3-5 animals per experiment were included in analysis, which resulted in large sample sizes of regions of interest.
Data exclusions	Imaging fields were discarded if fluorescent reporter expression was low or if the retina did not respond to light stimuli. In addition, low quality (noisy) fields were discarded. Additional quality filtering was performed to discard unsuccessful Gaussian Process predictions or noisy receptive field maps - these are discussed in detail in the Methods.
Replication	Datasets in the manuscript include at least 3 animals with a large number of samples per animal. All attempts at replication were successful.
Randomization	Stimuli were presented in a pseudorandom order and the order of presentation of different stimuli was varied. Location within the retina was randomly selected. No other randomization was used.
Blinding	Owing to the exploratory nature of our study, we did not use randomization and blinding of animals.

Reporting for specific materials, systems and methods

We require information from authors about some types of materials, experimental systems and methods used in many studies. Here, indicate whether each material, system or method listed is relevant to your study. If you are not sure if a list item applies to your research, read the appropriate section before selecting a response.

Materials & experimental systems	
n/a	Involved in the study
☒	☐ Antibodies
☒	☐ Eukaryotic cell lines
☒	☐ Palaeontology and archaeology
☐	☒ Animals and other organisms
☒	☐ Clinical data
☒	☐ Dual use research of concern

Methods	
n/a	Involved in the study
☒	☐ ChIP-seq
☒	☐ Flow cytometry
☒	☐ MRI-based neuroimaging

Animals and other research organisms

Policy information about studies involving animals; ARRIVE guidelines recommended for reporting animal research, and Sex and Gender in Research

Laboratory animals	We use laboratory mice of either sex. For glutamate imaging, we used either the ChAT-cre transgenic line (n = 3; JAX 006410, The Jackson Laboratory) or C57Bl/6 J (n = 7, JAX 000664) mice. For Ca2+ imaging, the ChAT-cre transgenic line was crossbred with the Cre-dependent green fluorescent reporter line Ai59D (n = 3; JAX 024105) or the ChAT-cre transgenic line (n = 3; JAX 006410, The Jackson Laboratory) was injected with a virus for expression of GCaMP8f. Mice were between 6 weeks and 3 months of age.
Wild animals	No wild animals were used in this study.
Reporting on sex	Animals of both sexes were used in this study and data from both sexes were combined. Due to low numbers of animals, we did not perform sex-specific analysis.
Field-collected samples	No field collected samples were used in the study.
Ethics oversight	All animal procedures were approved by the governmental review board (Regierungspräsidium Tübingen, Baden-Württemberg, Konrad-Adenauer-Str. 20, 72072 Tübingen, Germany) and performed according to the laws governing animal experimentation issued by the German Government.

Note that full information on the approval of the study protocol must also be provided in the manuscript.
